# Case Report: Giant mesenteric hemolymphangioma enveloping the ileum

**DOI:** 10.3389/fonc.2025.1557916

**Published:** 2025-06-18

**Authors:** Guan-Ying Yu, Qi Liu, Jing-Du Yan, Xiao-Yan Shi, Qing-Qing Yang, Bao-xuan Wang, Cheng-Zhen Li, Pei-ming Guo, Jie Jiao, Hou-Jun Zhang, Jing-Bo Shi, Lei Zhang

**Affiliations:** Department of Gastrointestinal Surgery, Central Hospital Affiliated to Shandong First Medical University, Jinan, Shandong, China

**Keywords:** hemolymphangioma, abdominal mass, laparoscopic surgery, gastrointestinal involvement, case report

## Abstract

Hemolymphangiomas are rare, benign tumors arising from lymphatic and vascular tissues, most commonly found in subcutaneous and soft tissues, with their occurrence in the gastrointestinal tract, especially the mesentery, being uncommon. Due to their heterogeneous imaging features, these tumors are often misdiagnosed as malignancies, particularly when located in the abdominal cavity. We report a case of a 16-year-old male presenting with abdominal pain, vomiting, and diarrhea for 7 days. Imaging revealed a large, heterogeneous mass in the right lower abdomen, initially suspected to be a malignant mesenteric tumor. An ultrasound-guided biopsy and immunohistochemistry confirmed the diagnosis of hemolymphangioma. The patient underwent a combined laparoscopic and open surgical approach, including en bloc resection of the tumor, along with a segment of the ileum and surrounding mesentery. Histopathological examination verified the presence of lymphatic and vascular components consistent with hemolymphangioma. The patient recovered uneventfully and showed no recurrence at a 3-month follow-up. Hemolymphangiomas, although rare, should be considered in the differential diagnosis of mesenteric tumors.

## Introduction

Hemolymphangiomas are rare, benign tumors originating from lymphatic and vascular structures, most commonly found in subcutaneous and loose connective tissues (1). Although they predominantly occur in the head, neck, and extremities, involvement of the gastrointestinal tract is exceedingly rare (2). Mesenteric hemolymphangiomas, particularly those involving the small intestine, present unique challenges in diagnosis and management due to their nonspecific symptoms and imaging findings, which can mimic malignant tumors (3). This report describes a case of a giant mesenteric hemolymphangioma in a 16-year-old male, notable for its unusual size and involvement of the ileum, which was encased and compressed by the tumor. Such cases are exceptional, with few documented in the literature.

## Case presentation

A 16-year-old male from China presented to the hospital with a 7-day history of abdominal pain, vomiting, and diarrhea. The abdominal pain was intermittent, localized to the right lower quadrant, and severe during episodes. It was accompanied by vomiting of gastric contents and diarrhea occurring four times daily. The patient had no prior medical or surgical history and denied smoking, alcohol consumption, or drug use. There was no family history of genetic disorders or malignancies. On physical examination, a palpable, firm mass with limited mobility and poorly defined borders was noted in the right lower quadrant. Bowel sounds were slightly hyperactive. Laboratory tests revealed normal levels of tumor markers, including CEA, CA19-9, and alpha-fetoprotein (AFP), but elevated CA125 at 36.6 U/L (reference range: 0–35 U/L) and fibrinogen at 4.33 g/L (reference range: 2–4 g/L).

**Figure 1:**
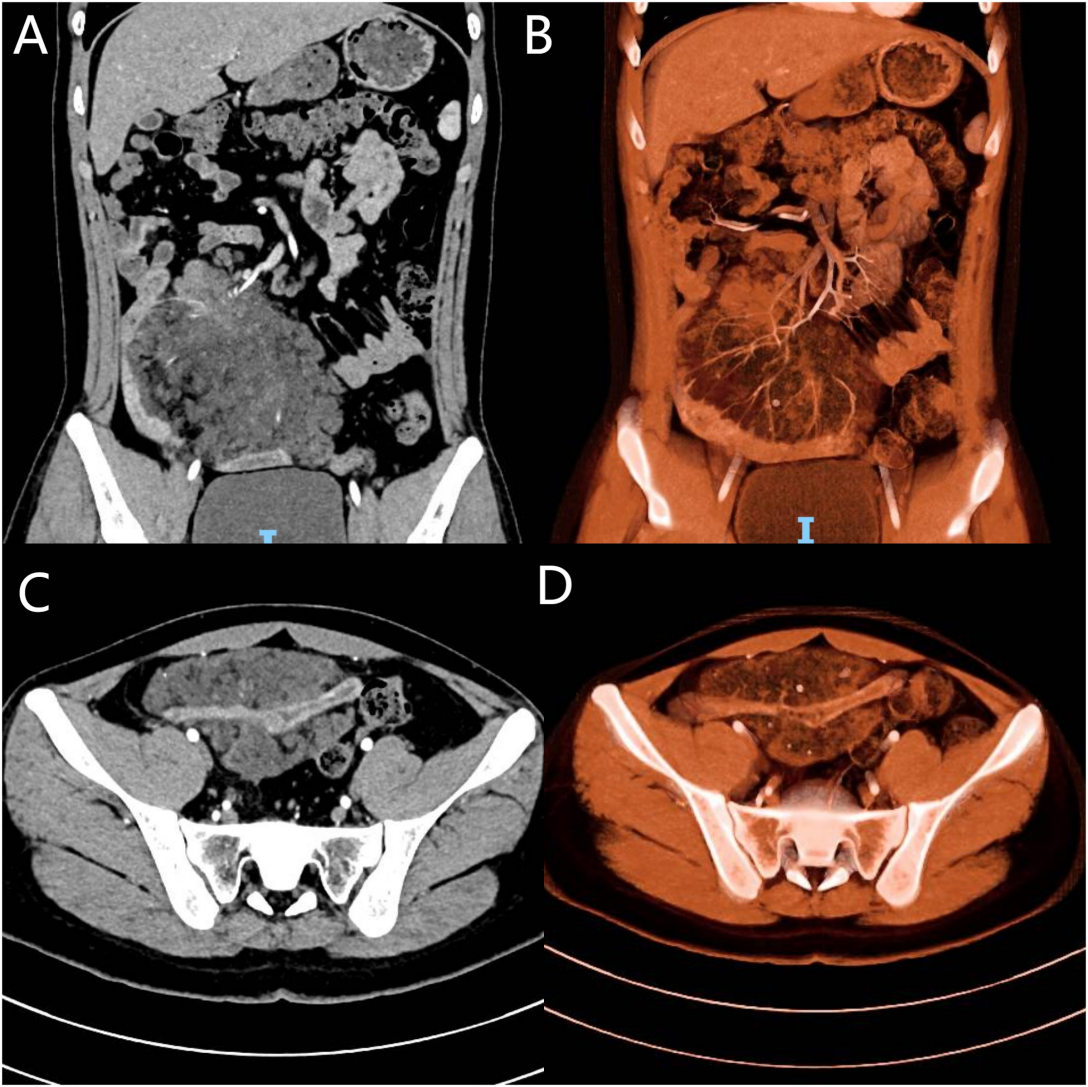
Contrast-enhanced CT and 3D reconstruction: **(A)** Coronal view showing a right lower abdominal mass originating from the mesentery. **(B)** Coronal 3D vascular reconstruction showing the tumor’s blood supply from the superior mesenteric artery. **(C)** Axial view showing the tumor encasing and invading the ileum. **(D)** Axial 3D reconstruction showing ileal involvement.

**Figure 2:**
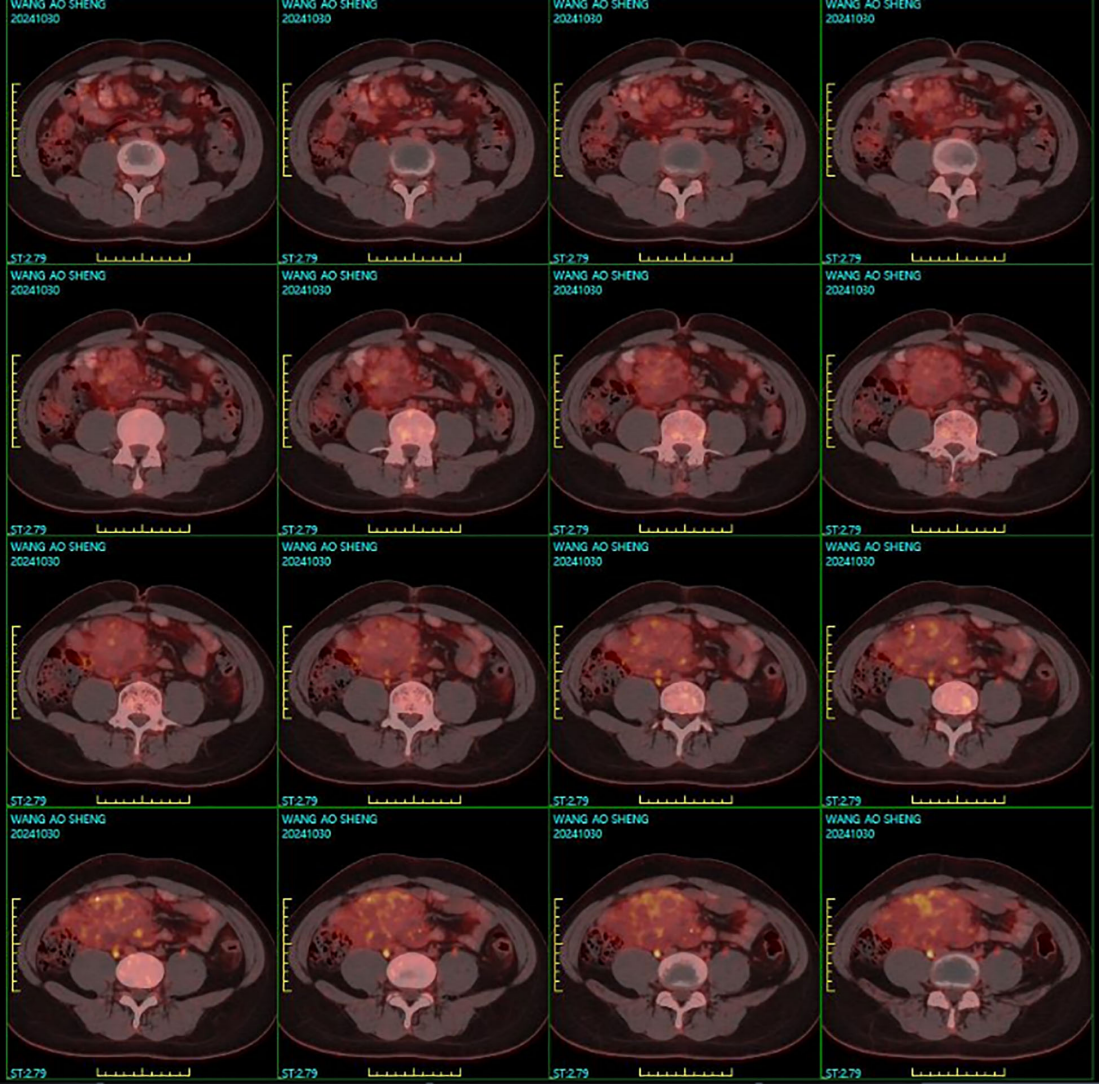
PET/CT imaging showing an irregular soft tissue mass in the mesenteric region of the right lower abdomen and pelvis. The lesion exhibits well-defined margins with mildly increased FDG uptake.

Imaging studies included abdominal ultrasonography and ultrasound-guided biopsy, which identified a substantial mass in the right lower abdominal and pelvic regions, suggesting a space-occupying lesion. Biopsy samples included skeletal muscle, adipose tissue, and fibrous stroma, with dysplastic-like changes and focal lymphocytic proliferation. Immunohistochemical analysis revealed positivity for CD34 (vascular marker) and D2-40 (lymphatic marker), with a low Ki-67 proliferative index of approximately 3%. Staining for CK and CR was negative, and CD68 and SMA showed limited positivity. Gastrointestinal endoscopy revealed no abnormalities. Abdominal contrast-enhanced computed tomography (CT) demonstrated a mixed-density lesion measuring 18 × 10.3 × 6.9 cm in the right lower abdomen, with significant heterogeneous enhancement and prominent vascularity. The mass had indistinct borders with the adjacent small bowel, suggesting mesenteric involvement. Multiple small lymph nodes were observed in the retroperitoneal, pelvic, and bilateral inguinal regions. Findings were highly suggestive of a malignant mesenteric tumor (**Figure 1**). Magnetic resonance imaging (MRI) revealed a large heterogeneous soft tissue mass (approximately 168 × 140 mm) with high signal intensity on DWI, reduced signal on ADC, and slightly hyperintense signals on T2WI. Delayed heterogeneous enhancement, with non-enhancing fibrous and fatty areas, was observed. The lesion contained multiple mesenteric arterial branches and demonstrated thickened peritoneum with areas of enhancement and nodular structures, raising strong suspicion for malignancy. Positron emission tomography-computed tomography (PET/CT) showed an irregular soft tissue mass in the mesenteric region of the right lower abdomen and pelvis. The mass demonstrated mildly increased FDG uptake, with a maximum SUV of 2.8, increasing to 3.2 on delayed imaging. Calcified nodules and adipose components were visible within the lesion, along with vasculature traversing the mass. Adjacent intestinal loops were compressed and displaced. Findings were suggestive of a mesenchymal tumor of mesenteric origin, with a hamartomatous lesion considered likely (**Figure 2**).

**Figure 3:**
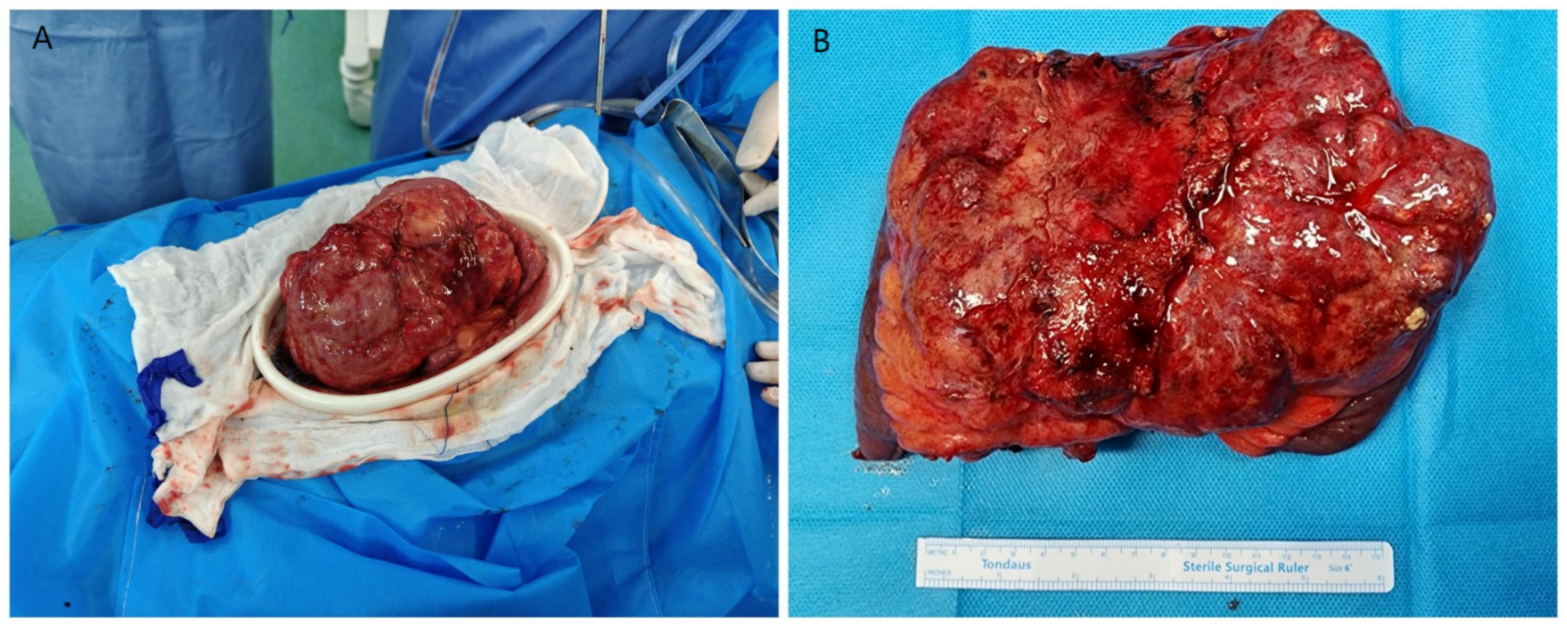
**(A)** Intraoperative exposure of the tumor during open surgery. **(B)** The completely resected tumor measuring 18 × 10 × 8 cm.

**Figure 4:**
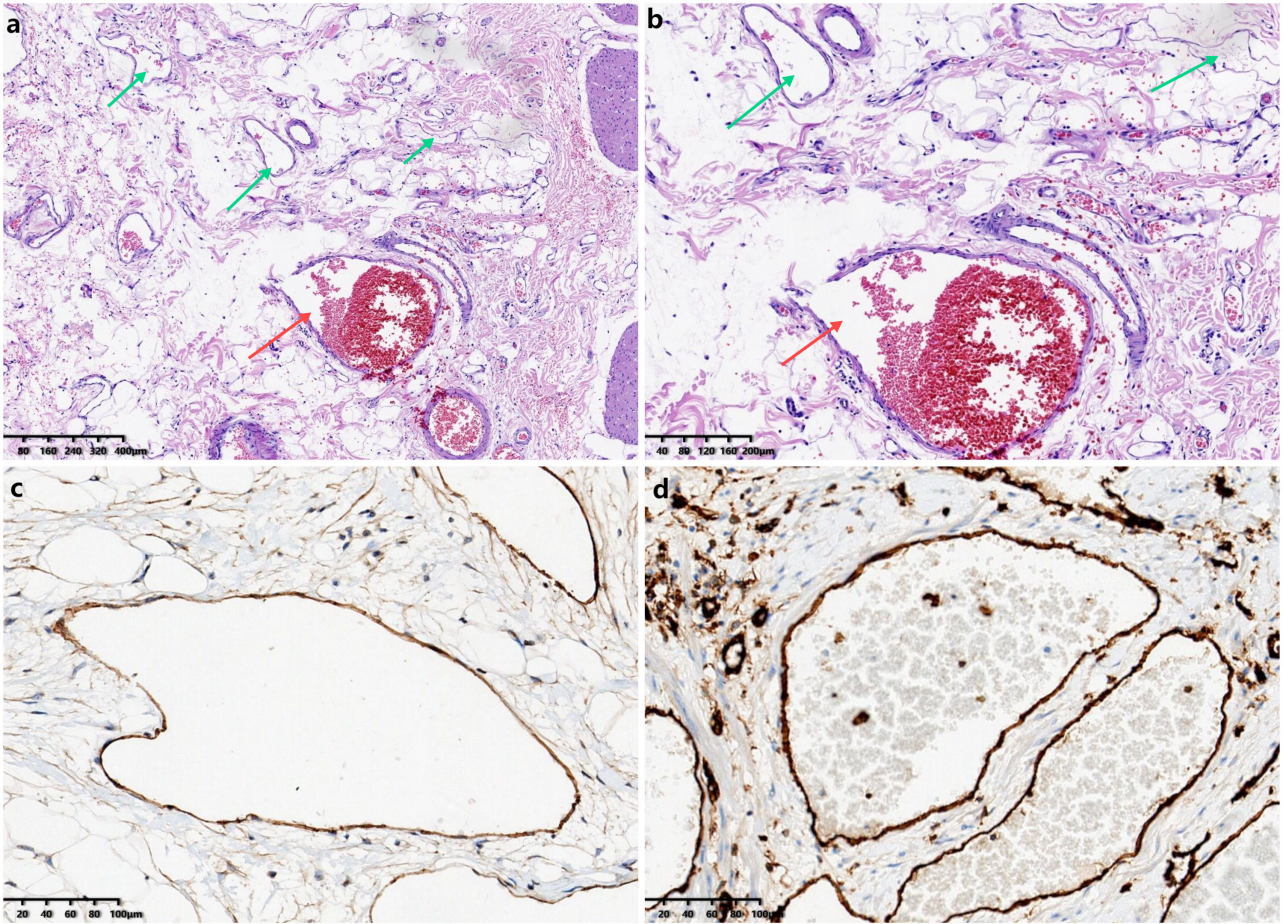
Histopathological examination and immunohistochemistry: **(a)** Hematoxylin and eosin staining (50×) showing predominantly lymphatic channels (green arrow) with vascular components (red arrow). **(b)** Hematoxylin and eosin staining (100×) revealing prominent vascular hemorrhage (red arrow). **(c)** Immunohistochemical staining(100×) demonstrating lymphatic channels positive for D2-40. **(d)** Immunohistochemical staining (100×) demonstrating vascular structures positive for CD34.

The patient underwent surgery using a combined laparoscopic and open approach. Laparoscopic exploration localized the tumor to the mesentery of the proximal ileum, where it encased and compressed the ileum and adhered densely to the surrounding structures. Due to the large size of the tumor, the dense adhesion with the surrounding structures, especially the fixation with the posterior peritoneal adhesion, and the limited space for laparoscopic operation, in order to ensure the complete resection of the tumor and reduce the collateral damage of the surrounding blood vessels and organs, it was decided to switch to open surgery. Adhesions were carefully separated to define tumor boundaries, followed by en bloc resection of the tumor, a segment of the ileum, and the surrounding mesentery (**Figure 3**). Mesenteric vessels were meticulously dissected and ligated. Multiple enlarged lymph nodes surrounding the tumor were excised. The small intestine was transected on both sides of the tumor using a stapler, and functional end-to-end anastomosis was performed to restore bowel continuity. The operation time was 2 hours and 8 minutes, and the intraoperative bleeding was about 20ml. Pathological examination confirmed the mass as a hemolymphangioma. Grossly, the tumor was dark red, irregular, and solid, partially encasing and adhering tightly to the jejunum without penetrating the intestinal wall. Microscopically, the tumor consisted predominantly of lymphatic channels with focal hemorrhagic vasculature, extending from the subserosa into the muscularis propria. Immunohistochemical analysis showed positivity for D2–40 and CD34, confirming lymphatic and vascular components, with a Ki-67 index of approximately 1% (**Figure 4**).

The patient had an uneventful recovery, defecation on the 2nd postoperative day, removal of the gastrointestinal decompression tube and initiation of enteral nutrition on the 3rd day, removal of the abdominal drainage tube on the 7th postoperative day, and was discharged on postoperative day 12. At the 6-month follow-up, there were no signs of recurrence, good eating and bowel movements and no additional therapy was required.

## Discussion

Hemolymphangiomas are rare, benign tumors originating from mesenchymal tissue, with a higher prevalence in children and adolescents, while cases in older adults are less frequently reported. These tumors typically develop in regions rich in loose connective tissue, such as the subcutaneous layers of the head, neck, and extremities (4). Involvement of the gastrointestinal system is unusual, with only a handful of cases documented in organs like the esophagus, stomach, pancreas, liver, and gallbladder (3, 5). The occurrence of hemolymphangiomas in the small intestine, particularly in the jejunum or ileum, as seen in this case, is even more uncommon. These tumors are classified into primary and secondary types. Primary hemolymphangiomas are believed to stem from congenital abnormalities in the venolymphatic system, likely due to developmental defects or blockages during embryogenesis. Secondary hemolymphangiomas are generally linked to lymphatic obstruction caused by trauma, surgery, or inflammation. Given the lack of any prior abdominal surgery, trauma, or infections in this patient, a primary hemolymphangioma is the most plausible diagnosis, consistent with its presumed congenital origin (6).

Hemolymphangiomas are typically asymptomatic in their early stages. As the tumor enlarges, it may compress adjacent organs, resulting in symptoms such as abdominal pain, bloating, or back pain (7). In severe cases, it can lead to gastrointestinal bleeding or obstruction. Large hemolymphangiomas may also present as palpable abdominal masses. In this case, the patient experienced abdominal pain, vomiting, and diarrhea without any other specific clinical manifestations, necessitating auxiliary diagnostic methods for an accurate diagnosis. Diagnostic imaging, including ultrasound, CT, and MRI, plays a key role in evaluating hemolymphangiomas (6, 8). On ultrasound, these tumors usually appear as irregular, cystic lesions with unclear borders, and often contain tubular anechoic structures separated by echogenic septa. CT imaging often shows a mass with varying density depending on the proportion of vascular components. If the vascular elements are prominent, the mass may appear slightly hyperdense on unenhanced CT, and show enhancement on contrast imaging (8). MRI typically reveals heterogeneous signal intensity on T1- and T2-weighted images, with areas of low signal intensity corresponding to fibrous or fatty tissue, and high signal intensity areas corresponding to fluid-filled lymphatic spaces (9). The imaging characteristics of hemolymphangiomas lack specificity, often complicating accurate diagnosis. In this case, imaging revealed a hemolymphangioma encasing and invading the ileum, which initially led to a suspicion of malignancy.

The diagnosis of mesenteric hemolymphangioma in the ileocecal region requires differentiation from cavernous hemangioma, simple lymphangioma, and lymphangioleiomyoma (7, 9). Cavernous hemangiomas are primarily composed of large, thin-walled vascular channels, often containing red blood cells, but lacking pinkish lymphatic fluid and lymphocytes (9). They are immunohistochemically positive for CD31 and CD34 but negative for D2-40. Simple lymphangiomas consist of irregular, thin-walled cavities filled with lymphatic fluid and lymphocytes, generally without red blood cells, and are strongly positive for D2-40. In contrast, lymphangioleiomyomas exhibit reticular or sinusoidal spaces lined by endothelial or spindle-shaped cells, surrounded by smooth muscle-like cells, and express markers such as HMB-45, Melan-A, and SMA, distinguishing them from hemolymphangiomas, which do not express HMB-45 or Melan-A (7).

Hemolymphangiomas are benign tumors arising from lymphatic and vascular structures that do not regress spontaneously. While slow-growing, they can disrupt organ function and, in the intestinal tract, may lead to bleeding, bowel obstruction, or intussusception (3, 7). Mesenteric involvement can further result in complications such as venous thrombosis. Their infiltrative growth pattern and local aggressiveness make conservative treatments like aspiration or laser ablation prone to high recurrence rates (8). Thus, complete surgical resection is the preferred treatment, tailored to the tumor’s size, location, and proximity to surrounding structures, with a focus on total excision while preserving adjacent organs and tissues. The recurrence rate after complete resection is low, ranging from 10% to 27%, whereas the recurrence rate after partial resection ranges from 50% to 100% (10). In the present case, a combined laparoscopic and open surgical approach was adopted, avoiding unnecessary exploratory laparotomy. Laparoscopy served both diagnostic and therapeutic roles, facilitating confirmation of preoperative imaging findings and enabling precise tumor resection under enhanced visualization. The expanded and clear surgical field offered by laparoscopy reduced the risk of missing residual disease and allowed for meticulous, accurate dissection. Ultrasonic scalpels and electrosurgical tools were predominantly employed during surgery, ensuring effective coagulation before vascular transection, which resulted in thorough hemostasis and minimal blood loss. After achieving partial tumor resection laparoscopically, the procedure transitioned to open surgery for the completion of the remaining steps, ensuring comprehensive removal of the tumor.

## Conclusion

Hemolymphangiomas often lack specific clinical symptoms, making advanced imaging techniques, such as CT and MRI, crucial for accurate diagnosis and treatment planning. Additionally, immunohistochemical markers like D2–40 and CD34 provide definitive pathological confirmation. While cases involving the small intestine are exceedingly rare, hemolymphangiomas should be considered in the differential diagnosis of unexplained and persistent gastrointestinal symptoms. For symptomatic cases, early surgical intervention is recommended. A combined laparoscopic and open surgical approach may offer advantages in selected cases by enabling precise tumor localization and resection, and can be considered as a viable option in complex abdominal procedures.

## Data Availability

The original contributions presented in the study are included in the article/supplementary material. Further inquiries can be directed to the corresponding author.
